# Prevalence and Risk Factors of Childhood Hypertension in Urban-Rural Areas of China: A Cross-Sectional Study

**DOI:** 10.1155/2020/2374231

**Published:** 2020-05-11

**Authors:** Xiaohua Liang, Lun Xiao, Yetao Luo, Jiapei Xu

**Affiliations:** ^1^Clinical Epidemiology and Biostatistics Department, Children's Hospital of Chongqing Medical University, Ministry of Education Key Laboratory of Child Development and Disorders, National Clinical Research Center for Child Health and Disorders, Key Laboratory of Pediatrics in Chongqing, China International Science and Technology Cooperation Center of Child Development and Critical Disorders, Chongqing, China; ^2^Disease Control and Prevention Center of Jiulongpo District, Chongqing, China

## Abstract

**Objective:**

The increased blood pressure level in children and adolescents is recognized as an essential predictor of adulthood cardiovascular disease. This study aimed to ascertain the prevalence and the urban-rural disparity of childhood hypertension in the southwest of China.

**Methods:**

Using stratified cluster sampling in urban and rural areas, a total of 13597 primary school children aged 6∼12 years living in the Southwest of China were included. The prevalence of hypertension was analyzed. The risk factors were collected by questionnaires, and the risk factors of childhood hypertension were analyzed by the logistic regression model.

**Results:**

The prevalence of hypertension was 13.75%, 9.02%, and 17.47% in total, urban, and rural children, respectively, and the urban-rural difference was 8.44% (95%CI: 7.32%, 9.56%). Children with obesity, maternal gestational hypertension, >10 months of breastfeeding, or low family income had a significantly increased prevalence of hypertension (29.4%, 20.00%, 16.31%, and 16.25%, respectively). Rural residence, intake of more pickle (in rural), maternal gestational hypertension (in urban), low birth weight (in rural), obesity, increased heart rate, and red blood cell counts were the risk factors of childhood hypertension. The adjusted *R*^2^ values were 13.61%, 23.25%, 10.88%, 11.12%, 12.23%, and 25.04% in the full models excluding and including serum indexes for total, urban, and rural children, respectively.

**Conclusions:**

The prevalence of childhood hypertension is significant in the Southwest of China and alarming in rural areas, which requires community intervention. Children living in rural areas combined with obesity, low social economic status, dietary imbalance, and abnormal lipid metabolism were associated with an increased risk of hypertension, and routine care programs should be conducted to prevent childhood hypertension.

## 1. Introduction

Elevated blood pressure level during childhood and adolescence is a recognized predictor of adulthood BP, leading to increased CVD disease in adults [1, 2]. It is imperative to study the prevalence and risk factors for childhood and adolescents' hypertension, especially in low social economic status (SES) population. Despite the urban-rural differentials in BP have decreased in China, the BP level of rural children is constantly higher than that of urban children in China [3]. As well known, obesity is a stronger risk factor of childhood hypertension [4], and the urban-rural disparity of hypertension prevalence could not be explained by obesity alone [3], as the prevalence of childhood obesity in urban areas was higher than that of rural counterparts [5]. Obviously, it indicates that the risk factors of hypertension between urban and rural areas are different and other potential factors could provide further explanation. Previous studies have shown that higher BP levels were associated with a sedentary lifestyle [6], high dietary sodium intake [7], elevated low-density lipoprotein cholesterol (LDL-C), and high total cholesterol (TC) than those of their counterparts [8, 9]. These influencing factors are different in urban and rural areas. The dietary pattern of malnutrition could increase the risk of hypertension [10], and the dietary pattern was different between urban and rural children. In addition, children living in the city reported higher levels of physical activity than those living in rural areas [11]. Moreover, children living in urban areas usually have higher education levels and SES compared to those living in rural areas in China.

The difference between urban and rural children in obesity, SES, dietary patterns, and physical activity was observed, and the prevalence of hypertension and the risk factors may vary between urban and rural areas. There are limited studies explored this relationship, and the present study will illustrate the prevalence of childhood hypertension and analyze the difference between urban and rural areas. This cross-sectional study with measures of anthropometric variables, dietary intake, physical activity, glycolipid indexes and blood cell composition measures, and perinatal factors in children provides an excellent opportunity to illustrate the differences of prevalence and the risk factors of hypertension between rural and urban areas in a large urban-rural population in Southwest of China. Furthermore, this study assesses to what extent these variables account for the level of variability in BP among rural and urban children aged 6–12 years.

## 2. Methods

### 2.1. Subjects

Two stages stratified (county and community) cluster sampling was used to choose participates. First, one urban county and one rural county were randomly selected from all urban and rural counties in Chongqing in Southwest of China, which represent urban and rural areas, respectively; second, two communities per county were randomly selected from all the communities of the two counties; and finally, all children living in the selected communities and studied in the local primary schools were included in the present study. A cross-sectional study was designed to assess the risk factors of childhood hypertension and their differences between urban and rural regions, with evaluations conducted in 2014. Inclusion criteria were as follows: (1) 6∼12 years old in 2014, (2) living in the survey areas for more than 6 months, (3) without serious diseases such as nephropathy, cardiovascular disease, and cancer, and (4) informed consent from both parents and children. This cross-sectional study planned to include 15 000 subjects, the rate of participate was 90.71%, and 13 597 samples were included in this study (in Supplementary Figure 1). The approval for this study was agreed by the Institutional Review Board at the Children's Hospital of Chongqing Medical University. Both children and parents provided informed consent. The teachers in the school submitted and collected the questionnaires.

### 2.2. Demographic Variables, Dietary Intake, and Physical Examination

Demographic variables, SES variables, prenatal variables, and dietary intake variables were collected, which were described in detail in previous publication [12]. Family history of parental obesity and hypertension was surveyed by questionnaires. In addition, physical activity (PA) was assessed by the self-reported activity categories (very heavy PA, heavy PA, medium PA, light PA, and sedentary lifestyle) and the duration of PA time per day, at weekday or weekend, spent on PA that was sufficient to “work up a sweat”. The average time per day of PA at weekday or weekend and overall PA time were used to analyze the children's PA status. Moreover, dietary intake was surveyed by the self-reported food intake frequency questionnaire, and the details of dietary intake can be found in a previous study [13].

Details of physical examination were introduced in a previous study [12]. A mobile medical ultrasonic machine (model WS-H300D) was used to measure height and weight, and BMI was calculated as weight/height^2^ (kg/m^2^). BP levels and heart rate were measured on three separate occasions with an OMRON arm-type electronic sphygmomanometer (HEM-7051). Children were diagnosed with hypertension if all three BP measurements met the criteria for hypertension, according the diagnose criteria of Chinese children [14]. Physical examinations and medical-history interviews were implemented by a pediatrician to exclude secondary hypertensive subjects.

### 2.3. Biochemical Indexes and Blood Cell Composition

A total of 5971 samples had biochemical index variables. Details of venous blood sample collection and measurement can be found in a previous study [12]. The serum glycolipid index was measured by an automatic biochemical analyzer (Mindray BS-800), and blood-cell composition was measured by automated hematology analyzers (Sysmex K-4500).

### 2.4. Diagnostic Criteria

The hypertension diagnostic criteria described by Mi Jie [15] were used in the present study, which were suitable for the growth characteristics of children in China. Hypertension was defined as mean clinic-measured SBP and/or DBP ≥95th percentile based on age, gender, and height percentiles. BP was measured by an electronic sphygmomanometer in this study. According to the sex-specific CDC BMI-for-age growth charts, overweight was diagnosed as 85th percentile ≤ BMI and BMI ≤ 95th percentile, and obesity was diagnosed as BMI ≥95th percentile [16].

### 2.5. Statistical Analyses

Normal distribution continuous variables of anthropometric variables, serum glycolipid indexes, and blood cell composition variables were reported as mean and standard deviation (SD), and Student's *t*-test was used to assess the differences between urban and rural areas. Skewed distribution continuous variables of dietary were expressed as median (interquartile range), and the Wilcoxon rank sum test was used to compare the difference between urban and rural areas. The prevalence of hypertension by categorical variables was reported as percentages of prevalence (%) and 95% confidence interval (CI), and the chi-squared test was used to test the difference between urban and rural areas. Moreover, the univariate logistic regression model was performed to analyze the relationship between hypertension and SES, perinatal measures, anthropometric measures, serum glycolipid indexes, blood cell composition, and dietary intake after adjusted age, gender, and height. Next, all of the significant variables were added to the multivariable logistic regression model to estimate their effects on childhood hypertension. Finally, all significant variables in the previous models were entered simultaneously to full model 1, and full model 2 added glycolipid and blood cell composition indexes based on full model 1.

Statistical analysis was performed using SAS 9.4 software (Copyright © 2016 SAS Institute Inc., Cary, NC, USA). A *p* value <0.05 was considered significant.

## 3. Results

### 3.1. General Characteristics

The general characteristics of the subjects are presented in Table 1. The mean age was 9.23 ± 1.76 years, and 51.92% (7060/13597) were males. Compared with children from urban areas, children living in rural areas had significantly elevated age, heart rate, FGB, TC, TG, HDL-C, LDL-C, white blood cell (WBC), red blood cell (RBC), hemoglobin (HGB), hematocrit (HCT), mean corpuscular volume (MCV), WBC small cell ratio (WSCR), WBC small cell count or lymphocyte count (WSCC), red blood cell distribution width (RDWSD), mean platelet volume (MPV), and the percent of living with grandparents, but significantly reduced BMI, weight, waist circumference, MCHC, PLT, WMCR, WMCC, WLCC, PA, and father with obesity (all *P* < 0.05, Table 1). There was significant difference in the percent of father's education, parental occupation, income, people live with child, birth weight, and breastfeeding between the urban and rural groups. In addition, in Supplementary Table 1, the average intake amount of fruit, fish, eggs, milk, bean food, nuts, mushrooms and algae food, and vegetables was lower for children living in rural areas than those living in urban areas (all *P* < 0.05), and the average intake of cereals and potatoes, pickle, and nutritional supplements was higher for children living in rural areas than its counterparts (*P* < 0.05).

### 3.2. Prevalence of Childhood Hypertension

Overall, the prevalence of hypertension in 6∼12-year-old children was 13.75%, and there was significant difference between urban and rural areas (8.44% (7.32%, 9.56%), *P* < 0.001) (Table 2). The prevalence was elevated in children with the characteristics of living in rural areas, younger age group, obesity, low family income, living with grandparents, no medical insurance, maternal gestational hypertension, low birth weight (LBW), >10 months of breastfeeding, parental obesity, and low PA (all *P* < 0.05) (Table 2). In the subgroup analyses by areas, the prevalence of hypertension in children with maternal gestational hypertension was markedly elevated in urban areas (*P* < 0.05) but not in rural areas (Table 2). The prevalence was higher for children with the characteristics of younger age group and overweight/obesity, in both urban and rural areas. In the subgroup analyses of age, obesity, income, living with grandparents, people live with child, medical insurance, birth weight, breast feeding, father with obesity, mother with obesity, and PA, the prevalence of hypertension in rural children was higher than those of urban children (all *P* < 0.05) (Table 2).

**Figure 1:**
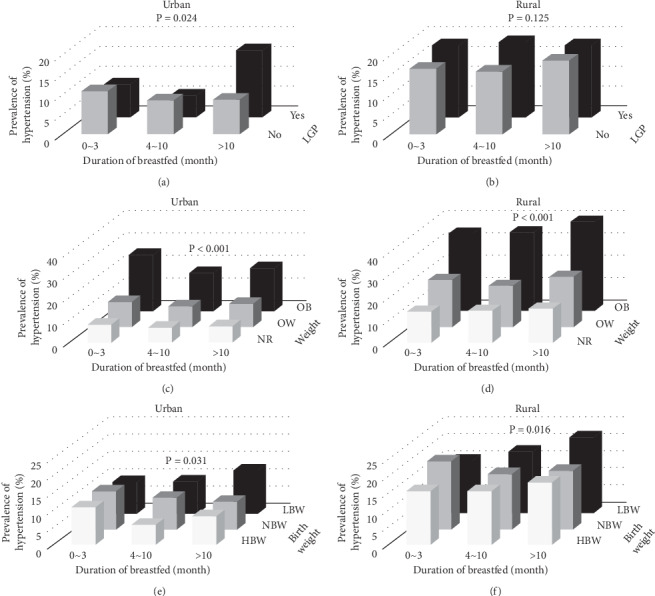
Prevalence of hypertension for children having different durations of breastfeeding combined with other characteristics. (a). Prevalence of hypertension for children combined with duration of breastfeeding and living with grandparents in urban regions. (b). Prevalence of hypertension for children combined with duration of breastfeeding and living with grandparents in rural regions. (c). Prevalence of hypertension for children combined with duration of breastfeeding and obesity in urban regions. (d). Prevalence of hypertension for children combined with duration of breastfeeding and obesity in rural regions. (e). Prevalence of hypertension for children combined with duration of breastfeeding and birth weight in urban regions. (f) Prevalence of hypertension for children combined with duration of breastfeeding and birth weight in rural regions.

**Figure 2:**
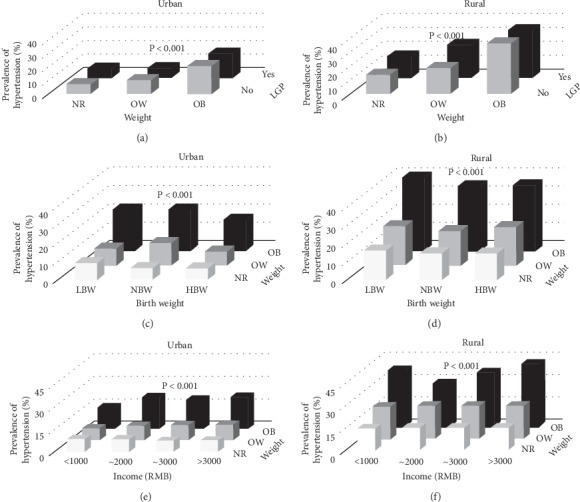
Prevalence of hypertension for children combined with two characteristics. (a). Prevalence of hypertension for children combined with present weight and living with grandparents in urban regions. (b). Prevalence of hypertension for children combined with present weight and living with grandparents in rural regions. (c). Prevalence of hypertension for children combined with birth weight and present weight in urban regions. (d). Prevalence of hypertension for children combined with birth weight and present weight in rural regions. (e). Prevalence of hypertension for children combined with income and present weight in urban regions. (f). Prevalence of hypertension for children combined with income and present weight in rural regions.

The prevalence of childhood hypertension was also analyzed in relation to pairs of the following variables: birth weight, duration of breastfeeding, family income, obesity, and living with grandparents. In Figure 1 and Figure 2, combinations of the above risk factors led to significantly higher risks for HTN in both urban and rural areas, it is shown in Figures 1 and 2 and Supplementary Tables 2–5. In Figure 1(a), children with >10 months of breastfeeding duration and living with grandparents had a significantly higher prevalence of hypertension than other groups (16.98%, *P* < 0.05) in urban regions but not in rural regions. In addition, obese children with 0∼3 months of breastfeeding duration or LBW (<3000 g) have the highest prevalence of hypertension in urban regions (25.19%, *P* < 0.001; 22.37%, *P* < 0.001) (Figures 1(c) and 2(c)), while obese children with >10 months of breastfeeding duration or LBW (<3000 g) have the highest prevalence of hypertension in rural regions (39.80%, *P* < 0.001; 43.80%, *P* < 0.001) (in Figure 1(d) and Figure 2(d)).

### 3.3. Using Univariate Model to Analyze the Risk Factors of Childhood Hypertension

The results of the univariate logistic regression model, adjusting age, sex, and height, illustrated that overweight/obesity, maternal gestational hypertension, >10 months of breastfeeding, maternal obesity, less weekday PA, and more pickle intake were risk factors for childhood hypertensive (all *P* < 0.05), whereas a paternal education level of 9∼12 years (ref. ≤ 9 y), high income, and increased birth weight were protective factors against childhood hypertensive (all *P* < 0.05) (Table 3). In the subgroup analyses, maternal gestational hypertension was a predictor of hypertension for urban children, while >10 months of breastfeeding and pickle intake effect the BP level only in rural areas (*P* < 0.05).

### 3.4. Effects of SES, Perinatal, Anthropometric, Biochemical, and Blood Cell Composition Measures on BP

A multivariable logistic regression model was used to analyze the relationship of SES, perinatal, anthropometric, glycolipid, and blood cell composition indexes with childhood hypertension (in Table 4). In the SES model, we found that income and medical insurance had a significant impact on BP (*P* < 0.05), explaining 0.58%, 1.13% and 0.57% of the variance of childhood hypertension in total, urban and rural areas, respectively.

In the perinatal measurement model, maternal gestational hypertension was a risk factor of childhood hypertension in urban regions but not in rural regions, while >10 months of breastfeeding was a predictor of hypertension (*P* < 0.001), especially for children in rural areas (*P*=0.052). The perinatal measurement model explained 0.64% and 0.28% of the variance of childhood hypertension in urban and rural areas, respectively.

Weight and heart rate were risk factors of elevated BP, whereas height was a protective factor against increased BP in both regions in the anthropometric measure model. Overall, the variance of BP explained in this model was almost equal between urban and rural areas (11.09% vs 11.73%).

In the serum glycolipid index model, FBG was a risk factor for BP in urban regions (*P* = 0.02) but not in rural regions, while HDL-C was a protective factor of hypertension only in rural regions (*P* < 0.05). In addition, TG was an important impact factor of hypertension both in urban and rural areas (all *P* = 0.001). This model explained a total of 1.19% and 4.09% of the variance of BP in urban and rural regions, respectively.

In the blood-cell composition model, RBC, PLT, RDWSD, and MPV were risk factors for high BP in total population, and RBC and PLT impact the BP level of children living in rural areas (*P* < 0.05). The variance of BP explained in the blood-cell composition model in rural areas was higher than that in urban areas (7.76% vs 3.76%).

### 3.5. Full Model to Analyze the Risk Factors of BP

The significant variables among the SES, perinatal, anthropometric, biochemical, and blood cell composition measure models and dietary variables were included in full model 1(in Table 5). Children living in rural areas have the increased odds ratio of hypertension compared with those in urban areas (2.21(1.93, 2.53), *P* < 0.001). Pickle intake and low birth weight (<3000 g) were significantly and positively associated with hypertension only in rural regions, but maternal gestational hypertension had significant increasing effects on the BP level only in urban regions. In addition, increased BMI and heart rate were risk factors for childhood hypertension both in urban and rural regions. Full model 1 could explain 13.61%, 10.88%, and 12.23% of the variance of childhood hypertension in total, urban, and rural areas, respectively.

Glycolipid index and blood cell compositions were added in full model 2 (in Table 5). In full model 2, rural residence, BMI, height, heart rate, and RBC were significant impact factors of childhood hypertension. In addition, maternal gestational hypertension was positively associated with hypertension, while height was negatively associated with BP in urban children (all *P* < 0.05). Moreover, BMI, heart rate, and RBC were positively associated with hypertension both in urban and rural areas (all *P* < 0.05). This model explained 23.25%, 11.12%, and 25.04% of the variance of hypertension in total, urban, and rural areas, respectively.

## 4. Discussion

The prevalence and the risk factors of childhood hypertension were assessed in this study. Our study included a variety of variables, such as perinatal, SES, lipid metabolism, and BCC, besides anthropometric variables, dietary variables, and physical activity in Southwest of China. Moreover, this study analyses the difference of the prevalence and risk factors between urban and rural areas.

The prevalence of childhood hypertension was 13.75%, and the prevalence in rural areas was nearly two times higher than that in urban areas, which is consistent with the results of other studies [3, 4]. However, the urban-rural disparity of the prevalence of childhood hypertension was controversial [3, 17]. Our study found the odds ratio (OR) of hypertension was 2.31 for residence in rural areas, which was lower than the results of 4.4 from Iranian [4], whereas the differential in BP cannot be explained only by obesity, as urban children have greater BMI and waist circumference than rural children in the present study, and the urban-rural disparity existed even after adjustment of BMI and other covariates, which suggested that the urban-rural disparity was an independent risk factor of childhood hypertension. Moreover, other potential factors may provide further opportunity to illustrate this urban-rural gap. Children in rural areas have increased heart rate, glycolipid levels, and breastfeeding, and decreased SES status and physical activities compared with those in urban areas. Besides, the dietary intakes and BCC measures were different between children in urban and rural areas in the present study. The high prevalence of childhood hypertension is alarming in rural areas and requires urgent intervention to focus on the main risk factors.

In this study, we found the prevalence was markedly higher in children of the age group 6∼7 compared with those in the middle age group in the present study, which was comparable with the results of another study, which found the prevalence in males of the age group 7 was higher than that in the age group 8∼10 [18], whereas most of the literature reported that the older age was a risk factor of hypertension in children [19, 20]. The results in this study may be attributed to two reasons: first, the diagnose criteria used in this study were made based on the data of 11 surveys from 2001 to 2009, and the modern lifestyle may impact the BP level, especially in the younger age group, as we found that the median BP levels of children aged 6∼7 years old in this study were significantly higher than those of the samples made the diagnose criteria (Supplementary Table 6); second, BP was measured by an OMRON arm-type electronic sphygmomanometer (HEM7051) in the present study, while mercury sphygmomanometers were used to measure BP for the reference samples, and it is more difficult to correctly hear phase 1 and phase 5 of Korotkoff sounds in younger age children than the middle age group using mercury sphygmomanometers.

In this study, we also found that children with overweight/obesity have higher prevalence of hypertension, which was in consistent with other research results [21, 22]. The odds ratio of developing hypertension in overweight and obese children were 1.5 and 3.6 times more than those with a normal weight and is consistent with the results of other studies [23]. The prevalence was 37.06% in children with obesity in rural areas, which suggested that overweight/obesity is one of the strongest predictors of hypertension in childhood [9, 21, 24], and health care intervention should be conducted for children who have obesity and live in rural areas.

Adverse perinatal measurements are associated with cardiovascular disease in adults, and increased BP might be one of the potential mechanisms [25, 26]. In this study, maternal gestational hypertension was a strong predictor of childhood hypertension in urban areas, which is consistent with other studies, indicating that genes and in-utero environment may account for the association. According to the literature [26–29], the present study found LBW was a major predictor of hypertension in rural children. However, some studies have reported that high birth weight is associated with an elevated risk of hypertension [30–32], while our study did not find such a correlation [33, 34]; this inconsistency may be induced by the different criteria for defining LBW and different characteristics of included samples. The correlation of breastfeeding with childhood hypertension was controversial [34–37]. The present study revealed that children with >10 months of breastfeeding had an elevated risk of hypertension, especially in rural children, whereas the literature [38, 39] reported that breastfeeding was not a predictor of blood pressure and cardiovascular disease in adults. The U-shaped relationship between duration of breastfeeding and hypertension was found [39], which agreed with our results that children with 4∼10 months of breastfeeding had the lowest prevalence of hypertension. There are 33.86% children with >10 months of breastfeeding, which may partly explain the high prevalence of hypertension in rural areas.

Low SES may be a predictor of hypertension. This study confirmed this concept in rural children. In the present study, children living in rural areas have low SES, which indicated as lower education of father and income, living with grandparents, more people live together, and uncovered by medical insurance. A long-term cohort study [3]observed that children whose fathers have a low education level was a predictor of increased blood pressure in young adults, which was consistent with our results. The study from D. Rose Ewald et al. [40] found that adolescents from low-income population have significantly higher prevalence of hypertension than those from high-income population. In addition, there was higher proportion of children in rural areas live with their grandparents compared with that of children in urban areas, which is another reason to cause the urban-rural disparity of the prevalence of hypertension, which agreed with a previous study [41] from Japanese children.

According to previous studies, unhealthy dietary intake [10] and micronutrient deficiency [12] are associated with childhood hypertension. Sodium intake is positively associated with increased BP and cardiovascular disease [10, 42] and is difficult to measure accurately in adulthood. Our study in children further confirmed this concept; we found that children in rural areas consumed more pickle than those living in urban and that the effect existed even after adjusting for other covariates. PA was inversely associated with the risk of hypertension [43], and Karatzi K. et al. [44] found that hypertension was positively associated with sedentary behaviors, and we found that this association existed specifically in weekday PA, suggesting that increase in the PA time for primary school children will control the blood pressure level of them.

In this study, we found that the relationship between blood-cell composition and hypertension was significant. Although sufficient data are not yet available on the association between red blood cell count and hypertension, hypertensive patients have been found to have elevated RBC and high uric acid [45], and we also found that children with hypertensive had increased RBC. Hypertension may trigger the activation of platelets, for which reason most antihypertensive agents also have antiplatelet effects, but the relationship between platelet count and the degree of hypertension remains unclear [46]. Accordingly, we found that increased PLT was associated with a higher risk of hypertension in childhood in the blood-cell composition model, suggesting that hypertension may promote the presence of spontaneous platelet aggregation and consequently predict vascular occlusions even in childhood.

The present study has some limitations. First, the prenatal variables were collected by a retrospective survey and the recall bias could not be eliminated. However, we checked perinatal medical records of mothers and reviewed the birth certificate of children to reduce the recall bias. Second, self-reported physical activities and dietary intake were used, which tends to be imprecise. Nevertheless, self-reported physical activities and dietary intake have been advocated in medical research. In addition, serum was drawn in only a subgroup of children. As the funding was limited, random sampling was used to control the sampling bias.

In conclusion, the prevalence of hypertension was serious in children living in rural areas, especially in those with the features of overweight/obesity, living with grandparents, maternal gestational hypertension, and more pickle diet. Our study emphasizes the importance of prevention in the impact of high blood pressure at the childhood stage, and it is of great important to screen and track hypertension from childhood and adolescents, especially in rural areas. It is essential to measure the blood pressure level of children at regular time intervals in primary and middle schools, which could improve the health of children and prevent the cardiovascular disease in adults.

## Figures and Tables

**Table 1:** General characteristics of children between urban and rural areas.

Variables	Total	Urban vs rural		
		Urban (*n* = 5995)	Rural (*n* = 7602)	*P*
*Anthropometric measures*				
Gender, male (*n* (%))	7060 (51.92%)	3098 (51.68%)	3962 (52.12%)	0.609
Age, y	9.24 ± 1.76	9.05 ± 1.76	9.39 ± 1.74	<0.001
BMI, kg/m2	17.35 ± 3.05	17.53 ± 3.02	17.22 ± 3.07	<0.001
Height, cm	134.51 ± 11.5	134.66 ± 11.44	134.39 ± 11.56	0.182
Weight, kg	32.01 ± 9.49	32.40 ± 9.53	31.7 ± 9.45	<0.001
Waist circumference, cm	58.12 ± 8.49	59.17 ± 8.73	57.3 ± 8.21	<0.001
Heart rate, n/min	95.28 ± 12.72	94.95 ± 12.71	95.54 ± 12.72	0.007
*Serum biochemical index*				
FBG, mmol/L	4.14 ± 0.62	3.94 ± 0.50	4.90 ± 0.46	<0.001
TC, mmol/L	3.49 ± 0.66	3.34 ± 0.57	4.10 ± 0.63	<0.001
TG, mmol/L	0.97 ± 0.58	0.96 ± 0.58	1.03 ± 0.57	<0.001
HDL-C, mmol/L	1.22 ± 0.27	1.19 ± 0.26	1.35 ± 0.28	<0.001
LDL-C, mmol/L	1.72 ± 0.54	1.56 ± 0.39	2.35 ± 0.60	<0.001
*Blood cell composition measures*				
WBC, *n* × 10^∧^9/L	7.12 ± 1.79	7.12 ± 1.77	7.16 ± 1.85	0.480
RBC, *n* × 10^∧^12/L	4.59 ± 0.39	4.52 ± 0.35	4.90 ± 0.40	<0.001
HGB, g/L	126.42 ± 9.98	124.31 ± 8.56	134.73 ± 10.82	<0.001
HCT, %	37.66 ± 3.11	36.74 ± 2.43	41.29 ± 2.78	<0.001
MCV, fL	81.91 ± 6.44	81.37 ± 5.98	84.01 ± 7.66	<0.001
MCH, pg	27.65 ± 1.95	27.64 ± 1.94	27.69 ± 1.96	0.390
MCHC, g/L	336.58 ± 14.98	338.99 ± 15.08	327.11 ± 10.06	<0.001
PLT, *n* × 10^∧^9/L	287.48 ± 67.49	288.53 ± 67.42	283.35 ± 67.63	0.020
WSCR, %	36.11 ± 8.25	35.59 ± 8.03	38.15 ± 8.80	<0.001
WMCR, %	7.05 ± 2.75	7.41 ± 2.87	5.64 ± 1.60	<0.001
WSCC, *n* × 10^∧^9/L	2.52 ± 0.66	2.47 ± 0.63	2.70 ± 0.73	<0.001
WMCC, *n* × 10^∧^9/L	0.51 ± 0.34	0.53 ± 0.28	0.44 ± 0.48	<0.001
WLCC, *n* × 10^∧^9/L	4.08 ± 1.49	4.12 ± 1.48	3.90 ± 1.50	<0.001
RDWSD, %	34.59 ± 4.82	33.40 ± 4.23	39.27 ± 4.09	<0.001
PDW, fL	13.47 ± 2.37	13.48 ± 2.32	13.39 ± 2.57	0.207
MPV, fL	10.68 ± 1.30	10.57 ± 1.32	11.11 ± 1.15	<0.001
P-LCR, %	32.67 ± 8.65	32.66 ± 8.86	32.69 ± 7.76	0.910
*Socioeconomic index*				
Father's education level, y				
∼9	6856 (51.75%)	2190 (37.80%)	4666 (62.58%)	<0.001
∼12	4915 (37.10%)	2787 (48.11%)	2128 (28.54%)	
∼15	1390 (10.49%)	755 (13.03%)	635 (8.52%)	
>15	88 (0.66%)	61 (1.05%)	27 (0.36%)	
Father's occupation				
Manager	933 (7.02%)	389 (6.70%)	544 (7.26%)	<0.001
Worker	4016 (30.21%)	1733 (29.84%)	2283 (30.49%)	
Technicist/researcher	571 (4.29%)	380 (6.54%)	191 (2.55%)	
Farmer	4593 (34.55%)	1686 (29.03%)	2907 (38.82%)	
Others	3182 (23.93%)	1619 (27.88%)	1563 (20.87%)	
Mother's occupation				
Manager	605 (4.55%)	241 (4.12%)	364 (4.88%)	<0.001
Worker	3538 (26.58%)	1666 (28.45%)	1872 (25.11%)	
Technicist/researcher	239 (1.80%)	132 (2.25%)	107 (1.44%)	
Farmer	4179 (31.40%)	2111 (36.05%)	2068 (27.74%)	
Others	4750 (35.68%)	1705 (29.12%)	3045 (40.84%)	
Income, RMB				
∼500	738 (6.25%)	92 (2.05%)	646 (8.83%)	<0.001
∼1000	1395 (11.82%)	235 (5.23%)	1160 (15.86%)	
∼2000	2327 (19.72%)	627 (13.96%)	1700 (23.25%)	
∼3000	2750 (23.30%)	1051 (23.41%)	1699 (23.24%)	
>3000	4592 (38.91%)	2485 (55.35%)	2107 (28.82%)	
Living with grandparents				
No	10060 (84.76%)	3992 (88.42%)	6068 (82.51%)	<0.001
Yes	1809 (15.24%)	523 (11.58%)	1286 (17.49%)	
People live with child				
1	583 (4.98%)	154 (3.43%)	429 (5.94%)	<0.001
2∼3	6114 (52.22%)	2595 (57.81%)	3519 (48.74%)	
4	5012 (42.80%)	1740 (38.76%)	3272 (45.32%)	
Medical insurance				
No	1790 (15.14%)	383 (8.50%)	1407 (19.24%)	<0.001
Yes	10030 (84.86%)	4124 (91.50%)	5906 (80.76%)	
*Perinatal measures*				
Gestational hypertension				
No	11299 (98.47%)	4366 (98.76%)	6933 (98.30%)	0.052
Yes	175 (1.53%)	55 (1.24%)	120 (1.70%)	
Birth weight, g				
∼3000	3181 (26.86%)	1133 (25.16%)	2048 (27.91%)	<0.001
∼3600	5051 (42.65%)	2058 (45.70%)	2993 (40.78%)	
>3600	3610 (30.48%)	1312 (29.14%)	2298 (31.31%)	
Breastfeeding, month				
0∼3	2845 (24.40%)	1420 (31.77%)	1425 (19.81%)	<0.001
4∼10	5555 (47.64%)	2223 (49.74%)	3332 (46.33%)	
>10	3261 (27.97%)	826 (18.48%)	2435 (33.86%)	
Father with obesity				
No	9839 (83.89%)	3703 (82.79%)	6136 (84.56%)	0.011
Yes	1890 (16.11%)	770 (17.21%)	1120 (15.44%)	
Mother with obesity				
No	10505 (89.57%)	4017 (89.73%)	6488 (89.48%)	0.670
Yes	1223 (10.43%)	460 (10.27%)	763 (10.52%)	
*Physical activity*				
Weekday, min/day				
∼10	6321 (53.41%)	2246 (49.87%)	4075 (55.59%)	<0.001
>10	5514 (46.59%)	2258 (50.13%)	3256 (44.41%)	
Weekend, min/day				
∼55	7782 (65.77%)	2795 (62.11%)	4987 (68.01%)	<0.001
>55	4051 (34.23%)	1705 (37.89%)	2346 (31.99%)	
Total, min/week				
∼150	6210 (52.59%)	2170 (48.32%)	4040 (55.21%)	<0.001
>150	5599 (47.41%)	2321 (51.68%)	3278 (44.79%)	

BMI, body mass index; FBG, fasting blood glucose; TC, total cholesterol; TG, triglyceride; HDL-C, high-density lipoprotein cholesterol; LDL-C, low-density lipoprotein cholesterol; WBC, white blood cell; RBC, red blood cell; HGB, hemoglobin; HCT, hematocrit; MCV, mean corpuscular volume; MCH, mean corpuscular hemoglobin; MCHC, mean corpuscular hemoglobin concentration; PLT, platelet; WSCR, WBC small cell ratio; WMCR, WBC middle cell ratio or mixed cell percent; WSCC, WBC small cell count or lymphocyte count; WMCC, WBC middle cell count or mixed cell count; WLCC, WBC large cell count or neutrophil count; RDWSD, red blood cell distribution width; PDW, platelet distribution width; MPV, mean platelet volume; P-LCR, platelet-large cell ratio.

**Table 2:** Prevalence of hypertension of children aged 6–12 years.

Variables	Total		Urban		Rural		Difference (rural vs urban)	*P*
	Prevalence	*P*	Prevalence	*P*	Prevalence	*P*		
Total	13.75% (13.17%, 14.34%)		9.02% (8.31%, 9.78%)		17.47% (16.62%, 18.34%)		8.44% (7.32%, 9.56%)	<0.001
*Anthropometric measures*								
Gender								
Male	13.50% (12.71%, 14.32%)	0.385	8.72% (7.75%, 9.76%)	0.388	17.24% (16.07%, 18.45%)	0.581	8.52% (6.98%, 10.06%)	<0.001
Female	14.01% (13.18%, 14.88%)		9.35% (8.32%, 10.47%)		17.72% (16.49%, 19.00%)		8.37% (6.73%, 10.00%)	<0.001
Age, y								
6	16.73% (14.92%, 18.66%)	<0.001	14.15% (11.83%, 16.72%)	<0.001	19.50% (16.75%, 22.49%)	<0.001	5.36% (1.67%, 9.04%)	0.004
7	15.66% (14.26%, 17.15%)		10.91% (9.23%, 12.79%)		20.35% (18.15%, 22.69%)		9.44% (6.61%, 12.26%)	<0.001
8	13.70% (12.32%, 15.17%)		7.30% (5.79%, 9.05%)		18.97% (16.85%, 21.24%)		11.67% (8.99%, 14.35%)	<0.001
9	11.68% (10.34%, 13.13%)		8.16% (6.47%, 10.12%)		14.41% (12.46%, 16.53%)		6.25% (3.58%, 8.91%)	<0.001
10	12.25% (10.90%, 13.70%)		7.31% (5.65%, 9.28%)		15.39% (13.49%, 17.44%)		8.08% (5.47%, 10.69%)	<0.001
11	13.28% (11.89%, 14.77%)		6.37% (4.84%,8.22%)		17.72% (15.71%, 19.87%)		11.35%(8.74%,13.96%)	<0.001
12	12.69% (10.35%, 15.33%)		8.24% (5.24%, 12.21%)		15.28% (12.11%, 18.91%)		7.04% (2.38%, 11.71%)	0.003
Obesity								
Normal	11.33% (10.70%, 11.99%)	<0.001	7.01% (6.24%, 7.84%)	<0.001	14.59% (13.65%, 15.56%)	<0.001	7.57% (6.35%, 8.80%)	<0.001
Overweight	15.50% (14.24%, 16.83%)		10.16% (8.63%, 11.85%)		20.04%(18.14%,22.04%)		9.88%(7.39%,12.37%)	<0.001
Obesity	29.40% (26.73%, 32.17%)		21.23% (17.84%, 24.93%)		37.06% (33.09%, 41.17%)		15.83% (10.58%, 21.09%)	<0.001
*Socioeconomic index*								
Father's education level, y^a^								
∼9	14.22% (13.40%, 15.07%)	0.256	8.36% (7.23%, 9.59%)	0.098	16.97% (15.91%, 18.08%)	0.465	8.62% (7.04%, 10.20%)	<0.001
∼12	13.06% (12.13%, 14.04%)		8.97% (7.93%, 10.09%)		18.42% (16.79%, 20.13%)		9.45% (7.49%, 11.41%)	<0.001
∼15	13.81% (12.04%, 15.74%)		10.73% (8.61%, 13.16%)		17.48% (14.60%, 20.66%)		6.75% (3.06%, 10.44%)	<0.001
>15	17.05% (9.87%, 26.55%)		14.75% (6.98%, 26.17%)		22.22% (8.62%, 42.26%)		7.47% (−10.56%, 25.5%)	0.417
Father's occupation^b^								
Manager	13.29% (11.18%, 15.64%)	0.110	11.31% (8.34%, 14.89%)	0.007	14.71% (11.84%, 17.97%)	0.006	3.39% (−0.94%, 7.73%)	0.125
Worker	14.82% (13.73%, 15.95%)		8.25% (7.00%, 9.65%)		19.80% (18.18%, 21.49%)		11.55% (9.46%, 13.63%)	<0.001
Technicist/researcher	13.84% (11.11%, 16.94%)		13.42% (10.16%, 17.27%)		14.66% (9.97%, 20.49%)		1.24% (−4.84%, 7.31%)	0.689
Farmer	13.89% (12.90%, 14.92%)		8.24% (6.98%, 9.66%)		17.17% (15.81%, 18.59%)		8.92% (7.02%, 10.82%)	<0.001
Others	12.60% (11.47%, 13.81%)		9.20% (7.84%, 10.72%)		16.12% (14.33%, 18.04%)		6.92% (4.62%, 9.22%)	<0.001
Mother's occupation^c^								
Manager	13.76% (11.11%, 16.77%)	0.096	10.79% (7.17%, 15.41%)	0.104	15.75% (12.15%, 19.91%)	0.268	4.87% (−0.54%, 10.28%)	0.078
Worker	14.32% (13.18%, 15.52%)		8.84% (7.51%, 10.31%)		19.16% (17.40%, 21.01%)		10.24% (8.00%, 12.49%)	<0.001
Technicist/researcher	16.74% (12.23%, 22.09%)		15.15% (9.51%, 22.43%)		18.69% (11.81%, 27.38%)		3.54% (−6.05%, 13.13%)	0.469
Farmer	12.74% (11.74%, 13.79%)		8.79% (7.61%, 10.09%)		16.73% (15.15%, 18.41%)		8.06% (6.06%, 10.07%)	<0.001
Others	14.26% (13.27%, 15.29%)		8.62% (7.33%, 10.06%)		17.40% (16.06%, 18.79%)		8.78% (6.89%, 10.68%)	<0.001
Income, RMB^d^								
∼500	16.26% (13.67%, 19.12%)	0.009	9.78% (4.57%, 17.76%)	0.880	17.18% (14.35%, 20.32%)	0.988	7.40% (0.67%, 14.13%)	0.031
∼1000	16.06% (14.17%, 18.09%)		8.51% (5.28%, 12.84%)		17.59% (15.44%, 19.90%)		9.08% (4.89%, 13.26%)	<0.001
∼2000	15.13% (13.69%, 16.65%)		8.29% (6.26%, 10.73%)		17.65% (15.86%, 19.54%)		9.35% (6.54%, 12.17%)	<0.001
∼3000	14.33% (13.04%, 15.69%)		8.85% (7.20%, 10.73%)		17.72% (15.93%, 19.62%)		8.87% (6.37%, 11.37%)	<0.001
>3000	13.00% (12.04%, 14.01%)		9.50% (8.37%, 10.72%)		17.13% (15.55%, 18.81%)		7.64% (5.66%, 9.62%)	<0.001
Living with grandparents^e^								
No	13.97% (13.29%, 14.66%)	0.039	9.27% (8.39%, 10.21%)	0.355	17.06% (16.12%, 18.03%)	0.099	7.79% (6.48%, 9.09%)	<0.001
Yes	15.81% (14.16%, 17.57%)		8.03% (5.85%, 10.70%)		18.97% (16.86%, 21.23%)		10.94% (7.78%, 14.11%)	<0.001
People live with child^f^								
1	15.44% (12.60%, 18.63%)	0.106	9.09% (5.06%, 14.78%)	0.687	17.72% (14.22%, 21.66%)	0.802	8.62% (2.82%, 14.43%)	0.004
2∼3	13.58% (12.73%, 14.46%)		8.82% (7.76%, 9.98%)		17.08% (15.85%, 18.36%)		8.25% (6.60%, 9.91%)	<0.001
4	14.86% (13.89%, 15.88%)		9.60% (8.25%, 11.08%)		17.67% (16.37%, 19.02%)		8.07% (6.16%, 9.97%)	<0.001
Medical insurance^g^								
No	13.83% (13.16%, 14.52%)	0.004	9.26% (8.39%, 10.19%)	0.209	17.01% (16.06%, 17.99%)	0.096	11.59% (8.28%, 14.91%)	<0.001
Yes	16.41% (14.72%, 18.20%)		7.31% (4.91%, 10.39%)		18.88% (16.87%, 21.02%)		7.79% (6.49%, 9.10%)	<0.001
*Perinatal measures*								
Gestational hypertension^h^								
No	14.02% (13.38%, 14.67%)	0.024	8.96% (8.12%, 9.84%)	0.018	17.21% (16.33%, 18.12%)	0.297	8.25% (7.02%, 9.48%)	<0.001
Yes	20.00% (14.34%, 26.70%)		18.18% (9.08%, 30.90%)		20.83% (13.96%, 29.20%)		2.65% (−9.87%, 15.17%)	0.678
Birth weight, g^i^								
∼3000	15.59% (14.35%, 16.90%)	0.026	9.89% (8.21%, 11.77%)	0.110	18.75% (17.08%, 20.51%)	0.149	8.86% (6.44%, 11.29%)	<0.001
∼3600	14.04% (13.09%, 15.03%)		9.52% (8.29%, 10.87%)		17.14% (15.81%, 18.54%)		7.62% (5.76%, 9.47%)	<0.001
>3600	13.35% (12.26%, 14.50%)		7.70% (6.31%, 9.28%)		16.58% (15.08%, 18.16%)		8.88% (6.79%, 10.98%)	<0.001
Breastfeeding, month^j^								
0∼3	13.74% (12.50%, 15.06%)	<0.001	10.42% (8.88%, 12.13%)	0.090	17.05% (15.13%, 19.11%)	0.084	6.63% (4.11%, 9.15%)	<0.001
4∼10	13.21% (12.33%, 14.13%)		8.28% (7.16%, 9.50%)		16.51% (15.26%, 17.81%)		8.23% (6.53%, 9.93%)	<0.001
>10	16.31% (15.06%, 17.63%)		9.20% (7.32%, 11.38%)		18.73% (17.20%, 20.33%)		9.53% (7.02%, 12.03%)	<0.001
Father with obesity^k^								
No	14.02% (13.36%, 14.70%)	0.149	8.99% (8.12%, 9.91%)	0.126	17.14% (16.23%, 18.08%)	0.263	8.42% (7.10%, 9.73%)	<0.001
Yes	16.19% (14.17%, 18.38%)		10.00% (7.41%, 13.11%)		19.92% (17.14%, 22.93%)		8.05% (4.91%, 11.20%)	<0.001
Mother with obesity^l^								
No	14.03% (13.35%, 14.73%)	0.040	8.78% (7.88%, 9.73%)	0.474	17.19% (16.26%, 18.16%)	0.055	8.15% (6.88%, 9.43%)	<0.001
Yes	15.29% (13.70%, 16.99%)		10.52% (8.44%, 12.90%)		18.57% (16.33%, 20.98%)		9.92% (5.98%, 13.86%)	<0.001
*Physical activity*								
Weekday, min/day^m^								
∼10	14.89% (14.02%, 15.79%)	0.028	9.26% (8.09%, 10.54%)	0.637	17.99% (16.82%, 19.20%)	0.141	8.73% (7.05%, 10.41%)	<0.001
>10	13.47% (12.58%, 14.40%)		8.86% (7.72%, 10.11%)		16.68% (15.41%, 18.00%)		7.82% (6.08%, 9.56%)	<0.001
Weekend, min/day^n^								
∼55	14.37% (13.59%, 15.17%)	0.560	8.91% (7.88%, 10.03%)	0.590	17.43% (16.38%, 18.51%)	0.900	8.52% (7.03%, 10.01%)	<0.001
>55	13.97% (12.92%, 15.08%)		9.38% (8.04%, 10.87%)		17.31% (15.80%, 18.90%)		7.92% (5.86%, 9.99%)	<0.001
Total, min/week^o^								
∼150	14.83% (13.96%, 15.74%)	0.050	9.17% (7.99%, 10.46%)	0.808	17.87% (16.70%, 19.09%)	0.247	8.70% (7.01%, 10.39%)	<0.001
>150	13.57% (12.69%, 14.50%)		8.96% (7.83%, 10.20%)		16.84% (15.57%, 18.17%)		7.88% (6.15%, 9.61%)	<0.001

^a^A total of 348 subjects having missing values in father's education level; ^b^A total of 302 subjects having missing values in father's occupation. ^c^A total of 286 subjects having missing values in mother's occupation. ^d^A total of 1795 subjects having missing values in parents' income. ^e^A total of 1728 subjects having missing values in living with grandparents. ^f^A total of 1888 subjects having missing values in people live with child. ^g^A total of 1777 subjects having missing values in medical insurance. ^h^A total of 2123 subjects having missing values in gestational hypertension. ^i^A total of 1755 subjects having missing values in birth weight. ^j^A total of 1936 subjects having missing values in breastfeeding. ^k^A total of 1868 subjects having missing values in father with obesity. ^l^A total of 1869 subjects having missing values in mother with obesity. ^m^A total of 1762 subjects having missing values in weekday activity. ^n^A total of 1764 subjects having missing values in weekend activity. ^o^A total of 1788 subjects having missing values in total activity

**Table 3:** Results of the univariate logistic model of the risk factors for childhood hypertension.

Variables^a^	Total			Urban			Rural		
	*β*	OR (95% CI)	*P*	*β*	OR (95% CI)	*P*	*β*	OR (95% CI)	*P*
Region (urban vs. rural)	−0.209	0.811 (0.707, 0.931)	0.003						
Obesity, ref. normal									
Overweight	0.404	1.497 (1.330, 1.686)	<0.001	0.446	1.563 (1.260, 1.938)	<0.001	0.395	1.484 (1.285, 1.714)	<0.001
Obesity	1.283	3.608 (3.095, 4.206)	<0.001	1.354	3.872 (2.988, 5.017)	<0.001	1.276	3.584 (2.946, 4.359)	<0.001
Education, ref. ≤9 y									
∼12	−0.137	0.872 (0.782, 0.972)	0.014	−0.003	0.997 (0.815, 1.221)	0.981	0.068	1.070 (0.936, 1.225)	0.322
∼15	−0.087	0.917 (0.775, 1.085)	0.313	0.166	1.181 (0.893, 1.562)	0.244	−0.017	0.983 (0.789, 1.225)	0.877
>15	0.156	1.169 (0.667, 2.049)	0.585	0.523	1.687 (0.814, 3.497)	0.159	0.306	1.358 (0.545, 3.383)	0.511
Father's occupation, ref. worker									
Manager	−0.141	0.869 (0.705, 1.071)	0.187	**0.326**	**1.386 (0.965, 1.990)**	**0.077**	**−0.384**	**0.681 (0.525, 0.883)**	**0.004**
Technicist/researcher	−0.108	0.898 (0.697, 1.157)	0.404	**0.498**	**1.646 (1.168, 2.318)**	**0.004**	**−0.377**	**0.686 (0.453, 1.039)**	**0.075**
Farmer	−0.078	0.925 (0.819, 1.045)	0.209	**0.003**	**1.003 (0.785, 1.282)**	**0.981**	**−0.179**	**0.836 (0.725, 0.963)**	**0.013**
Others	−0.194	0.824 (0.719, 0.944)	0.005	**0.106**	**1.112 (0.874, 1.414)**	**0.390**	**−0.251**	**0.778 (0.657, 0.923)**	**0.004**
Mother's occupation, ref. worker									
Manager	−0.051	0.950 (0.740, 1.220)	0.688	0.219	1.244 (0.799, 1.937)	0.333	−0.268	0.765 (0.563, 1.038)	0.086
Technicist/researcher	0.153	1.165 (0.819, 1.659)	0.396	0.531	**1.701 (1.024, 2.825)**	**0.040**	**−0.061**	**0.940 (0.570, 1.551)**	**0.810**
Farmer	−0.141	0.868 (0.761, 0.990)	0.035	−0.035	0.965 (0.769, 1.212)	0.762	−0.155	0.856 (0.727, 1.008)	0.063
Others	−0.002	0.998 (0.881, 1.131)	0.977	−0.015	0.985 (0.774, 1.254)	0.905	−0.117	0.890 (0.767, 1.033)	0.125
Income, ref. ≤500 RMB									
∼1000	−0.012	0.988 (0.775, 1.259)	0.920	−0.131	0.877 (0.383, 2.009)	0.757	0.026	1.027 (0.796, 1.325)	0.839
∼2000	−0.092	0.912 (0.727, 1.144)	0.428	−0.180	0.835 (0.396, 1.763)	0.637	0.028	1.029 (0.809, 1.308)	0.819
∼3000	−0.164	0.849 (0.679, 1.062)	0.151	−0.113	0.893 (0.433, 1.841)	0.759	0.019	1.019 (0.801, 1.296)	0.878
>3000	−0.297	0.743 (0.599, 0.921)	0.007	−0.081	0.922 (0.456, 1.866)	0.822	−0.027	0.973 (0.770, 1.231)	0.822
Living with grandparents (yes vs. no)	0.130	1.139 (0.991, 1.309)	0.066	−0.228	0.796 (0.568, 1.116)	0.186	0.120	1.127 (0.965, 1.316)	0.131
People live with child, ref. 2∼3									
1	0.165	1.179 (0.931, 1.494)	0.172	0.067	1.069 (0.606, 1.886)	0.817	0.051	1.052 (0.809, 1.370)	0.704
4	0.099	1.104 (0.992, 1.229)	0.070	0.069	1.071 (0.868, 1.322)	0.522	0.035	1.035 (0.913, 1.175)	0.589
Medical insurance (yes vs. no)	−0.209	0.811 (0.707, 0.931)	0.003	0.270	1.310 (0.878, 1.954)	0.187	−0.131	0.877 (0.755, 1.019)	0.087
Gestational hypertension (yes vs. no)	0.422	1.525 (1.049, 2.218)	0.027	**0.820**	**2.270 (1.132, 4.550)**	**0.021**	**0.232**	**1.261 (0.808, 1.970)**	**0.308**
Birth weight, 100g	−0.013	0.987 (0.976, 0.998)	0.018	−0.020	0.980 (0.959, 1.002)	0.076	−0.010	0.990 (0.978, 1.003)	0.137
Breastfeeding, ref. 4∼10 months									
0∼3	0.025	1.025 (0.898, 1.170)	0.714	0.223	1.250 (0.995, 1.572)	0.056	0.019	1.020 (0.863, 1.204)	0.819
>10	0.271	1.312 (1.161, 1.481)	<0.001	**0.157**	**1.170 (0.883, 1.551)**	**0.275**	**0.175**	**1.191 (1.038, 1.367)**	**0.013**
Father with obesity (yes vs. no)	0.088	1.092 (0.951, 1.254)	0.211	0.167	1.182 (0.913, 1.531)	0.205	0.072	1.075 (0.911, 1.268)	0.394
Mother with obesity (yes vs. no)	0.170	1.185 (1.007, 1.394)	0.041	0.087	1.091 (0.788, 1.510)	0.602	0.185	1.203 (0.995, 1.454)	0.057
*Physical activity*									
Weekday, min/day	−0.055	0.947 (0.899, 0.998)	0.040	−0.027	0.973 (0.879, 1.078)	0.603	−0.037	0.964 (0.906, 1.025)	0.235
Weekend, min/day	−0.023	0.977 (0.876, 1.091)	0.682	0.081	1.084 (0.879, 1.338)	0.450	−0.001	0.999 (0.877, 1.139)	0.993
Total, min/week	−0.096	0.908 (0.819, 1.008)	0.071	−0.023	0.977 (0.796, 1.200)	0.827	−0.058	0.944 (0.835, 1.067)	0.355
Dietary intake									
Pickle, g	0.002	1.002 (1.001, 1.004)	<0.001	0.001	**1.001 (0.997, 1.004)**	**0.963**	**0.002**	**1.002 (1.000, 1.003)**	**0.011**

^a^Adjusted age, gender, and height. The values in bold indicate that the variables have significant correlation with childhood hypertension only in one region, either urban or rural.

**Table 4:** Model of SES, perinatal, anthropometric, serum biochemical and blood cell composition for childhood hypertension.

Variables	Total			Urban			Rural		
	OR (95% CI)	*P*	R2	OR (95% CI)	*P*	R2	OR (95% CI)	*P*	R2
*SES model * ^a^			0.58			1.13			0.57
Education, ref. ≤9y									
∼12	0.983 (0.866, 1.115)	0.787		1.039 (0.800, 1.348)	0.776		1.137 (0.981, 1.317)	0.088	
∼15	0.981 (0.794, 1.212)	0.860		1.096 (0.757, 1.588)	0.627		1.087 (0.829, 1.426)	0.544	
>15	1.498 (0.824, 2.724)	0.185		1.755 (0.777, 3.968)	0.176		1.466 (0.583, 3.682)	0.416	
Father's occupation, ref. worker									
Manager	1.001 (0.754, 1.327)	0.999		1.574 (0.941, 2.633)	0.084		0.753 (0.533, 1.064)	0.108	
Technicist/researcher	1.103 (0.922, 1.319)	0.284		1.095 (0.744, 1.611)	0.646		1.088 (0.886, 1.335)	0.420	
Farmer	1.080 (0.789, 1.477)	0.631		1.996 (1.226, 3.248)	0.005		0.719 (0.451, 1.148)	0.167	
Others	0.952 (0.768, 1.179)	0.650		1.268 (0.835, 1.927)	0.265		0.894 (0.692, 1.155)	0.391	
Mother's occupation, ref. worker									
Manager	1.097 (0.806, 1.492)	0.557		1.062 (0.623, 1.809)	0.826		1.033 (0.704, 1.514)	0.869	
Technicist/researcher	1.062 (0.893, 1.262)	0.498		1.093 (0.795, 1.502)	0.586		1.055 (0.856, 1.302)	0.614	
Farmer	1.246 (0.825, 1.880)	0.296		1.147 (0.608, 2.163)	0.672		1.296 (0.748, 2.247)	0.355	
Others	1.022 (0.840, 1.244)	0.825		1.110 (0.751, 1.641)	0.600		0.966 (0.765, 1.219)	0.770	
Income, ref. ≤500 RMB									
∼1000	1.252 (0.995, 1.575)	0.055		1.146 (0.540, 2.430)	0.723		0.993(0.774,1.274)	0.957	
∼2000	1.272 (1.063, 1.523)	0.009		1.021 (0.616, 1.691)	0.936		1.046(0.856,1.278)	0.660	
∼3000	1.171 (1.007, 1.362)	0.041		0.948 (0.677, 1.327)	0.755		1.036(0.868,1.235)	0.696	
>3000	1.123 (0.974, 1.294)	0.111		0.991 (0.761, 1.291)	0.948		1.059 (0.891, 1.260)	0.515	
Living with grandparents (yes vs. no)	1.097 (0.949, 1.267)	0.211		0.777 (0.544, 1.109)	0.165		1.119 (0.952, 1.315)	0.172	
People live with child, ref. 2∼3									
1	1.094 (0.855, 1.399)	0.475		1.014 (0.550, 1.868)	0.965		1.028 (0.783, 1.348)	0.845	
4	1.107 (0.992, 1.235)	0.068		1.103 (0.888, 1.370)	0.377		1.054 (0.927, 1.198)	0.424	
Medical insurance (yes vs. no)	1.244 (1.080, 1.433)	0.002		0.882 (0.589, 1.320)	0.541		1.147 (0.983, 1.338)	0.081	
*Perinatal measure model * ^*b*^			0.48			0.64			0.28
Gestational hypertension (yes vs. no)	1.475 (1.008, 2.160)	0.045		**2.210 (1.102, 4.432)**	**0.025**		**1.209 (0.766, 1.907)**	**0.414**	
Birth weight, ref. 3000∼3600 g									
∼3000	1.142 (1.005, 1.298)	0.041		1.057 (0.825, 1.355)	0.661		1.122 (0.965, 1.304)	0.134	
>3600	0.923 (0.810, 1.052)	0.231		0.796 (0.615, 1.031)	0.084		0.948 (0.813, 1.104)	0.490	
Breastfeeding, ref. 4∼10 months									
0∼3	1.016 (0.887, 1.164)	0.820		1.253 (0.993, 1.582)	0.057		1.017 (0.857, 1.206)	0.851	
>10	1.265 (1.118, 1.433)	<0.001		1.119 (0.840, 1.489)	0.442		1.149 (0.999, 1.323)	0.052	
Mother with obesity (yes vs. no)	1.140 (0.962, 1.350)	0.130		1.040 (0.742, 1.458)	0.820		1.187 (0.974, 1.447)	0.089	
*Anthropometric measure model * ^*c*^			10.89			11.09			11.73
Weight, kg	1.040 (1.035, 1.044)	<0.001		1.046 (1.038, 1.055)	<0.001		1.038 (1.033, 1.043)	<0.001	
Height, cm	0.940 (0.932, 0.948)	<0.001		0.937 (0.921, 0.952)	<0.001		0.938 (0.929, 0.947)	<0.001	
Heart rate, n/min	1.089 (1.079, 1.099)	<0.001		1.087 (1.068, 1.106)	<0.001		1.096 (1.084, 1.108)	<0.001	
Total PA, ref. <150 min/week	0.902 (0.810, 1.005)	0.063		1.001 (0.810, 1.236)	0.998		0.925 (0.814, 1.051)	0.230	
*Serum biochemical index model * ^*d*^			8.86			1.19			4.09
FBG, mmol/L	1.771 (1.560, 2.012)	<0.001		1.256 (1.037, 1.521)	0.020		1.103 (0.840, 1.447)	0.480	
TC, mmol/L	0.803 (0.578, 1.114)	0.189		1.123 (0.775, 1.627)	0.539		1.318(0.684,2.537)	0.409	
TG, mmol/L	1.326 (1.166, 1.507)	<0.001		1.304 (1.114, 1.527)	0.001		1.504 (1.187, 1.905)	0.001	
HDL-C, mmol/L	1.114 (0.725, 1.711)	0.623		0.844 (0.502, 1.419)	0.523		0.420 (0.179, 0.986)	0.046	
LDL-C, mmol/L	2.262 (1.629, 3.140)	<0.001		1.124 (0.693, 1.823)	0.636		0.940 (0.507, 1.744)	0.845	
*Blood cell composition model * ^*e*^			12.04			3.76			7.76
RBC, *n* × 10^12/L	3.000 (1.716, 5.243)	<0.001		1.692 (0.790, 3.622)	0.176		2.657 (1.240, 5.692)	0.012	
HGB, g/L	1.012 (0.989, 1.035)	0.319		1.025 (0.958, 1.097)	0.468		1.007 (0.986, 1.029)	0.508	
HCT, %	1.051 (0.964, 1.147)	0.260		1.021 (0.812, 1.283)	0.862		1.017 (0.931, 1.112)	0.702	
MCV, fL	1.007 (0.980, 1.035)	0.611		0.998 (0.967, 1.030)	0.909		1.021 (0.976, 1.067)	0.370	
MCHC, g/L	1.010 (0.997, 1.023)	0.117		1.003 (0.976, 1.031)	0.834		0.999 (0.978, 1.019)	0.888	
PLT, *n*×10^9/L	1.004 (1.002, 1.005)	<0.001		1.002 (0.999, 1.004)	0.056		1.004 (1.001, 1.006)	0.004	
WSCR, %	0.997 (0.971, 1.024)	0.846		1.014 (0.971, 1.059)	0.523		0.988 (0.954, 1.022)	0.476	
WMCR, %	1.016 (0.951, 1.086)	0.638		1.046 (0.966, 1.133)	0.269		1.060 (0.911, 1.233)	0.450	
WSCC, *n*×10^9/L	1.261 (0.993, 1.601)	0.057		1.153 (0.784, 1.698)	0.469		1.249 (0.917, 1.702)	0.159	
WMCC, *n*×10^9/L	0.588 (0.259, 1.337)	0.205		0.572 (0.208, 1.570)	0.278		0.392 (0.067, 2.301)	0.300	
WLCC, *n*×10^9/L	1.057 (0.923, 1.209)	0.424		1.173 (0.965, 1.424)	0.108		1.073 (0.874, 1.317)	0.503	
RDWSD, %	1.056 (1.018, 1.096)	0.003		0.976 (0.924, 1.031)	0.388		1.015 (0.963, 1.069)	0.583	
MPV, fL	1.201 (1.111, 1.298)	<0.001		1.028 (0.922, 1.147)	0.618		1.140 (0.992, 1.308)	0.064	

SES, socioeconomic status; PA, physical activity; FBG, fasting blood glucose; TC, total cholesterol; TG, triglyceride; HDL-C, high-density lipoprotein cholesterol; LDL-C, low-density lipoprotein cholesterol; WBC, white blood cell; RBC, red blood cell; HGB, hemoglobin; HCT, hematocrit; MCV, mean corpuscular volume; MCHC, mean corpuscular hemoglobin concentration; PLT, platelet; WSCR, WBC small cell ratio; WMCR, WBC middle cell ratio or mixed cell percent; WSCC, WBC small cell count or lymphocyte count; WMCC, WBC middle cell count or mixed cell count; WLCC, WBC large cell count or neutrophil count; RDWSD, red blood cell distribution width; MPV, mean platelet volume. ^a^A total of 11265, 4294, 6971 subjects being included in SES model 1 of total, urban and rural, respectively. ^b^A total of 11171, 4328, and 6843 subjects being included in SES model 1 of total, urban, and rural areas, respectively. ^c^A total of 11795, 4491, and 7304 subjects being included in SES model 1 of total, urban, and rural areas, respectively. ^d^A total of 5955,4743,1212 subjects being included in SES model 1 of total, urban, and rural areas, respectively. ^e^A total of 5662, 4521, and 1141 subjects being included in SES model 1 of total, urban, and rural areas, respectively.

**Table 5:** Full model of risk factors for childhood hypertension.

Variables	Total			Urban			Rural		
	OR (95% CI)	*P*	R2	OR (95% CI)	*P*	R2	OR (95% CI)	*P*	R2 (%)
*Full model 1 * ^a^			13.61			10.88			12.23
Region (urban vs. rural)	**2.208 (1.926, 2.531)**	**<0.001**							
Income, ref. ≤500 RMB									
∼1000	1.052 (0.804, 1.377)	0.710		1.128 (0.424, 3.001)	0.809		1.037 (0.783, 1.373)	0.799	
∼2000	1.035 (0.804, 1.332)	0.790		1.029 (0.416, 2.542)	0.951		1.040 (0.798, 1.356)	0.771	
∼3000	1.004 (0.781, 1.290)	0.974		1.024 (0.422, 2.482)	0.958		1.004 (0.769, 1.310)	0.976	
>3000	0.950 (0.744, 1.213)	0.682		1.016 (0.426, 2.420)	0.972		0.920 (0.709, 1.194)	0.532	
Medical insurance (yes vs. no)	0.901 (0.774, 1.048)	0.177		1.173 (0.771, 1.784)	0.457		0.861 (0.730, 1.015)	0.075	
Total PA, ref. <150 min/week	0.929 (0.829, 1.041)	0.202		0.927 (0.746, 1.153)	0.497		0.920 (0.805, 1.053)	0.226	
Pickle, g	**1.002 (1.001, 1.003)**	**0.025**		1.001 (0.996, 1.004)	0.877		**1.002 (1.001, 1.004)**	**0.010**	
*Perinatal measures*									
Gestational hypertension (yes vs. no)	**1.615 (1.083, 2.407)**	**0.019**		**2.461 (1.203, 5.036)**	**0.014**		1.380 (0.857, 2.222)	0.186	
Birth weight, ref. 3000∼3600 g									
∼3000	**1.152 (1.005, 1.321)**	**0.042**		1.060 (0.814, 1.381)	0.664		**1.188 (1.013, 1.394)**	**0.035**	
>3600	0.932 (0.812, 1.070)	0.315		0.839 (0.642, 1.097)	0.201		0.966 (0.821, 1.135)	0.671	
Breastfeeding, ref. 4∼10 months									
0∼3	1.067 (0.923, 1.232)	0.382		1.206 (0.945, 1.538)	0.133		0.991 (0.827, 1.187)	0.918	
>10	1.124 (0.984, 1.284)	0.085		1.043 (0.770, 1.413)	0.786		1.134 (0.977, 1.317)	0.097	
*Anthropometric measures*									
BMI	**1.184 (1.161, 1.208)**	**<0.001**		**1.151 (1.109, 1.194)**	**<0.001**		**1.199 (1.171, 1.227)**	**<0.001**	
Height, cm	0.992 (0.983, 1.002)	0.107		0.994 (0.976, 1.012)	0.514		0.992 (0.981, 1.003)	0.148	
Heart rate, n/min	**1.040 (1.036, 1.045)**	**<0.001**		**1.047 (1.038, 1.056)**	**<0.001**		**1.037 (1.032, 1.043)**	**<0.001**	
*Full model 2 * ^b^			23.25			11.12			25.04
Region (urban vs. rural)	**2.310 (1.667, 3.205)**	**<0.001**							
Income, ref. ≤500 RMB									
∼1000	1.610 (0.898, 2.886)	0.328		1.021 (0.314, 3.325)	0.970		1.723 (0.873, 3.401)	0.374	
∼2000	1.544 (0.896, 2.662)	0.385		1.077 (0.362, 3.209)	0.814		1.619 (0.848, 3.090)	0.537	
∼3000	1.574 (0.921, 2.691)	0.261		1.105 (0.378, 3.233)	0.676		1.658(0.876,3.136)	0.423	
>3000	1.373 (0.811, 2.322)	0.836		0.960(0.334,2.760)	0.625		1.492 (0.799, 2.789)	0.921	
Medical insurance (yes vs. no)	0.841 (0.636, 1.112)	0.224		1.009 (0.632,1.613)	0.969		0.734 (0.509, 1.059)	0.098	
Total PA, ref. <150 min/week	0.989 (0.814, 1.200)	0.908		0.907(0.703, 1.170)	0.451		1.102 (0.811, 1.498)	0.534	
Pickle, g/day	1.114 (0.965, 1.287)	0.140		1.038 (0.830, 1.298)	0.742		1.177 (0.965, 1.435)	0.108	
*Perinatal measures*									
Gestational hypertension (yes vs. no)	1.939 (0.966, 3.891)	0.062		**2.535 (1.117, 5.753)**	**0.026**		1.055 (0.326, 3.421)	0.929	
Birth weight, ref. 3000∼3600 g									
∼3000	1.008 (0.794, 1.280)	0.759		1.057 (0.777, 1.437)	0.425		0.960 (0.657, 1.405)	0.607	
>3600	0.948 (0.753, 1.195)	0.604		0.880 (0.644, 1.203)	0.303		1.106 (0.772, 1.583)	0.469	
Breastfeeding, ref. 4∼10 months									
0∼3	1.255 (0.993, 1.586)	0.416		1.144 (0.857, 1.526)	0.615		1.436 (0.951, 2.167)	0.425	
>10	1.312 (1.035, 1.663)	0.166		1.133 (0.806, 1.595)	0.723		1.514 (1.075, 2.134)	0.156	
*Anthropometric measures*									
BMI, kg/m^2^	**1.146 (1.112, 1.181)**	**<0.001**		**1.155 (1.102, 1.211)**	**<0.001**		**1.138 (1.092, 1.185)**	**<0.001**	
Height, cm	**0.987 (0.978, 0.997)**	**0.009**		**0.975 (0.962, 0.988)**	**<0.001**		1.006 (0.991, 1.021)	0.448	
Heart rate, n/min	**1.046 (1.039, 1.054)**	**<0.001**		**1.043 (1.033, 1.054)**	**<0.001**		**1.051 (1.038, 1.064)**	**<0.001**	
*Biochemical index measures*									
FBG, mmol/L	1.080 (0.890, 1.311)	0.434		1.048 (0.820, 1.341)	0.706		1.207 (0.862, 1.689)	0.273	
TC, mmol/L	0.940 (0.781, 1.132)	0.516		0.966 (0.753, 1.239)	0.785		0.859 (0.647, 1.141)	0.294	
HDL-C, mmol/L	1.196 (0.783, 1.828)	0.408		1.185 (0.670, 2.096)	0.560		1.157 (0.606, 2.210)	0.658	
RBC, *n*×10^12/L	**1.946 (1.409, 2.687)**	**<0.001**		**1.742 (1.139, 2.664)**	**0.010**		**2.095 (1.239, 3.544)**	**0.006**	
HGB, g/L	1.006 (0.994, 1.018)	0.348		1.010 (0.992, 1.028)	0.274		0.999 (0.983, 1.016)	0.942	
WSCR, %	1.005 (0.994, 1.017)	0.366		1.012 (0.996, 1.028)	0.128		0.996 (0.978, 1.014)	0.668	
MPV, fL	1.017 (0.939, 1.101)	0.679		1.043 (0.945, 1.151)	0.408		0.954 (0.803, 1.096)	0.502	

PA, physical activity; BMI, body mass index; FBG, fasting blood glucose; TC, total cholesterol; HDL-C, high-density lipoprotein cholesterol; RBC, red blood cell; HGB, hemoglobin; WSCR, white blood cell small cell ratio; MPV, mean platelet volume. ^a^A total of 11014, 4265, and 6749 subjects being included in full model 1 of total, urban, and rural areas, respectively, and adjusted age and gender. ^b^A total of 4265, 3257, and 1008 subjects being included in full model 2 of total, urban, and rural areas, respectively, and adjusted age and gender. The values in bold indicate that the variables have significant correlation with childhood hypertension.

## Data Availability

The data used to support the findings of this study are included in the article. Requests for access to these details and data should be made to Xiaohua Liang (e-mail: xiaohualiang@hospital.cqmu.edu.cn; liangxiaohua666@sina.com).

## Supplementary Materials

Supplementary Materials Supplementary Figure 1: the flowchart of samples included. Supplementary Table 1: dietary intakes, g/day. Supplementary Table 2: the prevalence of hypertension combined with duration of breastfeeding and other risk factors. Supplementary Table 3: the prevalence of hypertension combined with birth weight and other risk factors. Supplementary Table 4: the prevalence of hypertension combined with weight and LGP. Supplementary Table 5: the prevalence of hypertension combined with duration of breastfed and other risk factors. Supplementary Table 6: comparison of the median of BP in the present study with the P50 of the diagnose criteria samples.
