# Supplementary material for: Prevalence and Risk Factors of Childhood Hypertension in Urban-Rural Areas of China: A Cross-Sectional Study
Source: Int J Hypertens. 2020 May 11;2020:2374231. doi: 10.1155/2020/2374231 (PMC7240786; doi:10.1155/2020/2374231)
Supplement: Supplementary Materials — Supplementary Figure 1: the flowchart of samples included. Supplementary Table 1: dietary intakes, g/day. Supplementary Table 2: the prevalence of hypertension combined with duration of breastfeeding and other risk factors. Supplementary Table 3: the prevalence of hypertension combined with birth weight and other risk factors. Supplementary Table 4: the prevalence of hypertension combined with weight and LGP. Supplementary Table 5: the prevalence of hypertension combined with duration of breastfed and other risk factors. Supplementary Table 6: comparison of the median of BP in the present study with the P50 of the diagnose criteria samples. [file 2374231.f1.pdf]

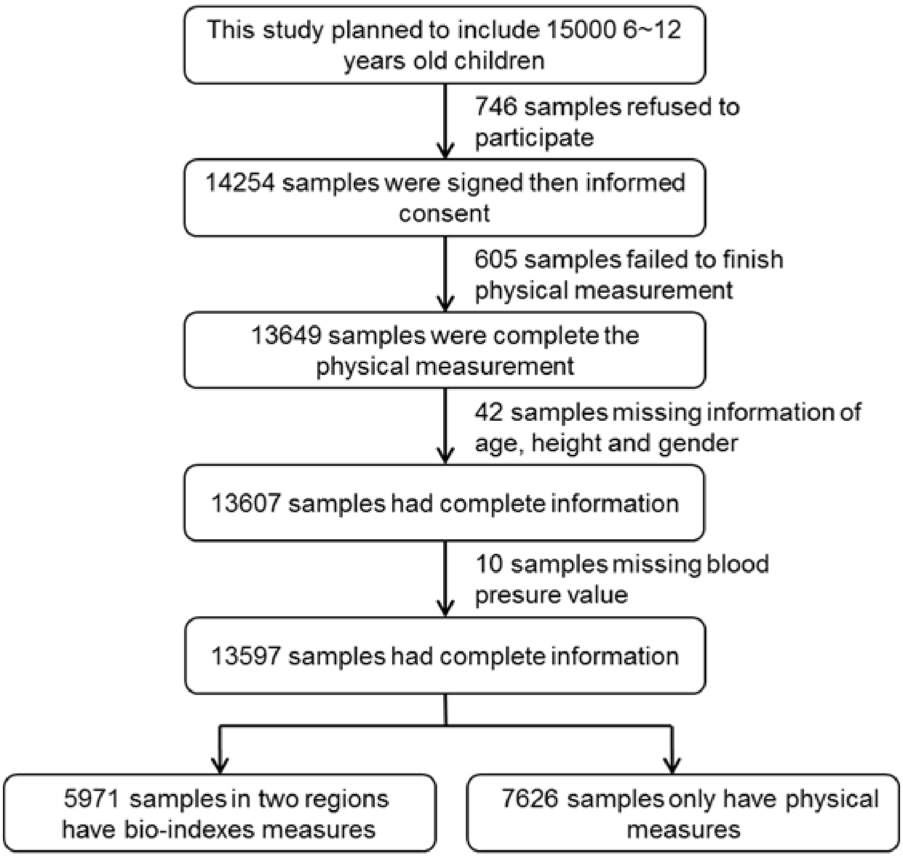

Supplementary Figure 1. The flow chart of samples included

Supplementary table 1. Dietary intakes, g/day

Variables	Total	Urban vs Rural		
		Urban (n=5995)	Rural (n=7602)	P
**Mean ±STD, g/day**				
Cereals and potatoes	150(100,250)	150(100,250)	150(100,250)	0.006
Vegetables	150(100,250)	150(100,250)	150(100,250)	<0.001
Fruit	150(71,250)	150(71,250)	142.86(71,250)	0.004
Red meat	100(50,150)	100(50,150)	100(42,150)	<0.001
Poultry	35(14,71)	35(14,71)	35(14,71)	0.089
Fish	16(5,35)	16(7,35)	14(4,35)	<0.001
Eggs	50(16,100)	50(21,100)	50(14,100)	<0.001
Milk	250(150,250)	250(190,280)	250(142,250)	<0.001
Bean food	28(14,71)	28(14,71)	28(10,71)	0.736
Nuts	11(2,35)	14(3,35)	8(1,35)	<0.001
Mushrooms and algae food	8(1,33)	13(3,33)	8(1,33)	<0.001
Oils	50(40,100)	50(50,100)	50(35,100)	0.925
Pickle	7(1,21)	5(1,14)	7(1,25)	<0.001
Nutritional supplements	0(0,5)	0(0,1)	0(0,8)	<0.001
Beverage	14(1,50)	14(1,33)	14(0,66)	<0.001
**Mean ±STD, g/day**				
Cereals and potatoes	204.83±158.52	192.61±136.66	212.42±170.26	<0.001
Vegetables	222.84±193.31	229.25±188.16	218.81±196.39	0.004
Fruit	190.45±179.43	188.43±166.44	191.70±187.00	0.324
Red meat	115.63±110.11	118.91±104.72	113.56±113.33	0.010
Poultry	60.28±83.51	58.58±75.81	61.36±88.00	0.074
Fish	38.27±75.38	36.02±64.64	39.66±81.26	0.007
Eggs	66.60±75.19	67.44±70.36	66.08±78.01	0.327
Milk	252.99±154.28	260.56±152.25	248.33±155.33	<0.001
Bean food	59.18±91.55	53.44±75.73	62.71±99.89	<0.001
Nuts	31.65±58.52	28.29±49.45	33.71±63.36	<0.001
Mushrooms and algae food	29.16±62.90	26.43±53.05	30.84±68.19	<0.001
Oils	74.60±61.08	72.21±52.68	76.07±65.69	<0.001
Pickle	19.18±35.68	14.15±29.08	22.27±38.87	<0.001
Nutritional supplements	18.71±64.05	10.24±39.38	23.92±74.83	<0.001
Beverage	47.66±100.38	36.50±81.15	54.52±110.00	<0.001

Supplementary table 2. The prevalence of hypertension combined duration of breastfed and other risk factors

Variables	Duration of breastfed, month			χ^2	P
	<3	4～10	>10		

Urban	LGP†					
	No	128(10.88)	172(8.62)	67(8.68)	12.976	0.024
	Yes	20(8.33)	12(5.41)	9(16.98)		
	Weight‡					
	NR	73(8.01)	96(6.66)	39(7.24)	82.902	<0.001
	OW	42(11.11)	54(9.23)	22(10.48)		
	OB	33(25.19)	34(17.26)	15(19.48)		
	Birth weight, g §					
	~3000	39(9.20)	48(9.56)	25(12.63)	16.944	0.031
	~3600	67(11.11)	99(9.16)	29(8.01)		
	>3600	42(10.85)	36(5.70)	22(8.40)		
	Income, RMB♯					
	<1000	5(6.17)	17(10.90)	6(6.98)	14.837	0.19
	～2000	13(7.26)	26(9.22)	12(7.41)		
	～3000	32(10.03)	41(7.98)	19(9.64)		
	>3000	98(11.89)	99(7.87)	38(10.11)		
Rural	LGP††					
	No	184(16.68)	441(15.96)	391(18.79)	8.622	0.125
	Yes	59(18.32)	107(19.04)	64(18.23)		
	Weight‡‡					
	NR	136(13.74)	329(14.13)	263(15.24)	187.379	<0.001
	OW	67(21.00)	141(18.19)	113(22.24)		
	OB	40(34.48)	80(35.09)	80(39.80)		
	Birth weight, g §					
	~3000	73(15.50)	169(18.09)	137(22.20)	18.785	0.016
	~3600	105(19.77)	231(16.11)	170(17.07)		
	>3600	65(15.48)	149(15.57)	146(18.02)		
	Income, RMB♯					
	<1000	52(16.51)	120(16.37)	136(18.92)	7.653	0.744
	～2000	44(14.97)	135(17.22)	113(19.45)		
	～3000	64(19.57)	133(16.48)	95(18.34)		
	>3000	82(17.15)	161(16.31)	108(18.03)		

LGP: Live with grandparents, NR: Normal weight, OW: Overweight, OB: Obesity. Missing data: † 1537 missing data, ‡ 1526missing data, § 1544 missing data, ♯1561 missing data, †† 419 missing data, ‡‡ 410 missing data, §§ 432 missing data, ♯♯ 460 missing data.

Supplementary table 3. The prevalence of hypertension combined birth weight and other risk factors

Variables		Birth weight, g			χ²	P
		~3000	~3600	>3600		
Urban	Income, RMB†					
	<1000	10(10.64)	7(5.98)	11(9.73)	8.695	0.650
	～2000	13(7.69)	26(9.67)	12(6.49)		
	～3000	24(9.38)	47(9.25)	21(7.50)		
	>3000	63(10.52)	116(10.05)	57(7.86)		
	Weight‡					
	NR	70(8.95)	88(6.64)	50(6.17)	91.671	<0.001
	OW	25(9.09)	66(12.18)	27(7.48)		
	OB	17(22.37)	42(22.11)	24(17.14)		
	LGP §					
	No	101(10.15)	176(9.65)	90(7.82)	5.298	0.381
	Yes	11(8.15)	20(8.70)	11(7.01)		
Rural	Income, RMB††					
	<1000	93(17.68)	112(15.86)	110(19.33)	12.034	0.361
	～2000	85(18.12)	125(18.52)	89(16.24)		
	～3000	92(20.67)	126(18.03)	82(14.91)		
	>3000	112(18.95)	147(16.46)	99(16.10)		
	Weight‡‡					
	NR	241(15.98)	301(14.58)	201(12.74)	204.946	<0.001
	OW	90(21.48)	128(18.55)	110(20.99)		
	OB	53(43.80)	84(35.29)	70(35.71)		
	LGP §§					
	No	304(18.23)	435(17.15)	293(15.88)	8.518	0.130
	Yes	79(20.90)	76(16.89)	88(19.47)		

LGP: Live with grandparents, NR: Normal weight, OW: Overweight, OB: Obesity. Missing data: † 1526 missing data, ‡ 1492 missing data, § 1503 missing data, †† 316 missing data, ‡‡ 263 missing data, §§ 272 missing data.

Supplementary table 4. The prevalence of hypertension combined weight and LGP

Variables		Weight			χ²	P
		NR	OW	OB		
Urban	LGP†					
	No	188(7.21)	107(10.46)	75(20.72)	79.222	<0.001
	Yes	22(6.79)	12(7.74)	8(18.18)		
Rural	LGP‡					
	No	591(14.03)	270(19.38)	174(37.50)	197.732	<0.001
	Yes	153(16.04)	58(24.27)	33(35.48)		

LGP:Live with grandparents.Missing data: † 1480 missing data.‡ 248 missing data.

Supplementary table 5. The prevalence of hypertension combined duration of breastfed and other risk factors

Variables		Income, RMB				χ^2	P
		<1000	～2000	～3000	>3000		
Urban	LGP†						
	No	26(9.35)	43(7.89)	86(9.33)	214(9.66)	4.913	0.671
	Yes	3(6.12)	9(11.25)	7(5.60)	22(8.24)		
	Weight‡						
	NR	21(8.61)	26(6.24)	49(7.08)	113(7.26)	77.926	<0.001
	OW	5(8.06)	15(9.49)	28(10.22)	71(10.43)		
	OB	3(14.29)	11(21.15)	16(18.82)	52(21.05)		
Rural	LGP §						
	No	231(16.75)	243(17.32)	258(18.27)	299(16.36)	10.399	0.167
	Yes	84(19.72)	56(18.98)	42(14.74)	61(22.18)		
	Weight♯						
	NR	200(14.71)	184(15.23)	177(15.17)	181(12.93)	209.056	<0.001
	OW	71(21.45)	82(21.81)	77(19.01)	98(19.07)		
	OB	44(38.26)	34(29.31)	47(37.01)	82(42.49)		

LGP: Live with grandparents,NR: Normal weight, OW: Overweight, OB: Obesity. Missing data: † 1514 missing data, ‡ 1505 missing data, § 299 missing data, ♯ 290 missing data.

Supplementary table 6. Compare the Median of BP in present study with the P50 of the diagnose criteria samples

Gender	Age, year	Height, cm	SBP			DBP		
			P50*	Mean	Median	P50*	Mean	Median
Male	6	<114	93	98.40	98.33	57	62.97	62.67
		114~116	94	96.69	96.67	57	60.43	60.33
		117~119	95	99.01	99.33	58	60.20	59.33
		120~123	96	100.57	100.50	58	61.94	62.00
		124~126	97	101.19	101.67	59	61.79	61.33
		127~130	98	103.44	103.00	59	62.84	62.67
		≥130	99	106.04	106.00	60	65.72	65.00
Male	7	<118	94	98.39	98.17	58	61.32	61.67
		118~120	95	99.81	99.67	58	62.19	62.50
		121~123	96	100.06	100.00	59	61.66	61.33
		124~127	97	101.18	100.67	59	62.09	61.67
		128~131	98	102.55	102.83	60	62.86	62.17
		132~135	100	105.18	105.00	61	63.87	63.00
		≥136	100	106.24	107.33	62	63.58	63.67
Female	6	<113	92	95.29	95.17	57	61.88	61.17
		113~114	92	96.04	96.00	57	60.89	60.33
		115~118	93	97.07	96.50	57	61.79	61.33
		119~121	94	98.00	97.00	58	61.79	62.33
		122~125	95	101.15	100.33	58	64.57	63.67
		126~128	96	100.38	100.00	59	63.71	62.67
		≥129	97	105.65	105.67	59	64.23	63.00
Female	7	<116	93	96.09	96.00	57	60.86	61.00
		116~118	93	97.29	97.17	57	61.91	61.33
		119~122	94	98.37	98.33	58	62.60	62.17
		123~126	95	98.88	99.33	59	62.50	61.67
		127~130	96	100.55	100.00	59	63.03	62.67
		131~133	97	101.12	100.67	60	62.50	61.83
		≥134	98	104.88	104.33	61	64.94	65.33

*Reference:FAN Hui YY-k MJ. Updating blood pressure references for Chinese children aged 3-17 years. Chin J Hypertens. 2017; 05:8
